# Targeting c-MET by Tivantinib through synergistic activation of JNK/c-jun pathway in cholangiocarcinoma

**DOI:** 10.1038/s41419-019-1460-1

**Published:** 2019-03-08

**Authors:** Kai Wei, Mao Li, Margot Zöller, Meng Wang, Arianeb Mehrabi, Katrin Hoffmann

**Affiliations:** Department of General, Visceral, and Transplantation Surgery, Ruprecht-Karls University, Heidelberg, Germany

## Abstract

Clinical treatment options for human cholangiocarcinoma (CC) are limited. c-MET, a high-affinity receptor for hepatocyte growth factor (HGF), is deregulated in many cancers. Its role in cholangiocarcinogenesis remains unclear. In current study, 23 corresponding tumor- and non-tumor tissues, taken from patients with intrahepatic (iCC) and perihilar cholangiocarcinoma (pCC), who underwent liver resection, were analyzed. The relationship of clinicopathological features and c-MET, as well as c-jun N-terminal kinase (JNK) was evaluated. The anti-tumor effects of Tivantinib, a small-molecule inhibitor with potent activity against the c-MET kinase, was investigated in three human CC cell lines, namely HUCC-T1, TFK-1, and EGI-1. In comparison with the results obtained in non-tumor tissue samples, c-MET was overexpressed in 91.3 % of tumor tissues (*p* < 0.01). The JNK expression was higher in tumor tissue compared with the corresponding non-tumor tissue sample in 17.4% patients (*p* < 0.01). The inhibition of aberrant c-MET expression in human CC cell lines was achieved by blocking the phosphorylation of c-MET with Tivantinib. Notable losses in cell viability and colony-forming capability were detected (*p* < 0.01). Synergistic activation of the JNK/c-jun pathway was demonstrated after Tivantinib treatment. Knockdown of the JNK by siRNA or competitive binding of c-MET receptor by stimulation with HGF-antagonized anti-tumor effects of Tivantinib was observed. Our data suggest that inhibition of c-MET could be a possible alternative approach for the treatment of human CC, for which Tivantinib may an effective inhibitor. The synergistic activation of the JNK/c-jun pathway contributed to the elevated apoptosis in CC cells via treatment with Tivantinib.

## Introduction

Untreated cholangiocarcinoma (CC) is one of most invasive malignancies with high mortality^1–4^. Most patients are diagnosed at an advanced stage, for which radical surgical resection is not feasible. The combination of Gemcitabine and Cisplatin is the only first-line palliative treatment for those patients and has limited benefits^5–8^.

The pro-tumorigenic function of c-MET, a high-affinity receptor of the hepatocyte growth factor (HGF), has a critical role in many solid tumors, including human CCs^9–16^. c-MET activates multiple downstream signaling pathways such as the phosphtidyl inositol 3-kinase (PI3K)/AKT/mammalian target of rapamycin (mTOR) pathway, the mitogen activated protein kinase (MAPK) pathway, and the STAT pathway, and is also involved in cell proliferation, differentiation, survival, mortality, and movement^13,17–19^. The aberrant expression of c-MET was recently considered as a potential target and biomarker in malignant tumors^20–22^. Although overexpression of c-MET has been described in patients with CC and in a mouse xenograft CC model, the precise function of c-MET signaling in cholangiocarcinogenesis still remains unclear^15,16,23,24^. The aim of this study was to explore the expression of c-MET in corresponding non-tumor and tumor tissues from CC patients, and its relationship with numerous clinicopathological factors. Tivantinib, a small-molecule kinase inhibitor with potent activity against c-MET, was investigated as an alternative therapeutic approach for CC in vitro.

## Methods

### Human tissue and immunofluorescence histochemistry

Twenty-three corresponding tumor- and non-tumor tissues were collected from patients with intrahepatic (iCC) and perihilar CC (pCC), who underwent liver resection. Clinicopathological characteristics of the patients are shown in Table 1. Tissue slides (7 μm) were soaked in 100 μL of goat serum blocking solution for 1 h after being washed twice with Tris-buffered saline with Tween20 (TBST) buffer for 5 min each. The slides were incubated overnight at 4 °C with the primary antibody at a concentration of 1 µg/mL. Cytokeratin 19 (CK19), which is normally expressed in the lining of the gastroenteropancreatic and hepatobiliary tracts, was applied in immunofluorescence histochemistry to distinguish the biliary duct system from other liver cells^25,26^. After three washes in phosphate-buffered saline (PBS), the tissue sections were incubated with 100 μL of secondary antibody in a dark, humid chamber at room temperature for 1 h. Finally, 100 μL of 4′,6-diamidino-2-phenylindole (DAPI) solution (Sigma-Aldrich, Munich, Germany) was introduced into each tissue area for 10 min before being counterstained with Mayer’s hematoxylin for 10 s, dehydrated in ethanol, and mounted. Immunohistochemistry was examined using a Zeiss Axiovert 40 CFl microscope. This study was carried out with the patients’ informed consent and approval from the local ethics committee. The approval number is 159/2002 and followed the guidelines stated in the Declaration of Helsinki.

**Table 1:** Clinicopathologic features of patients with cholangiocarcinoma

Characteristic	Group	*N* = 23	Ratio, %
Age	< 60	7	30.40%
	≥ 60	15	65.60%
Position	Intrahepatic	14	60.90%
	Perihilar	9	39.10%
TNM stage	0	0	0%
	I	7	30.50%
	II	5	21.70%
	III	6	26.10%
	IV	5	21.70%
Histologic grade	Well differentiated (G1)	3	13.00%
	Moderately differentiated(G2)	12	52.20%
	Poorly differentiated (G3)	8	34.80%
Surgical approach	R0	14	60.90%
	R1	9	39.10%
c-MET high expression	Tumor tissue	21	91.30%
	Non-tumor tissue	2	8.70%
JNK high expression	Tumor tissue	4	17.40%
	Non-Tumor tissue	16	69.60%

According to UICC 1st ed, 2018			
UICC stage			
TNM staging for intrahepatic bile duct tumors (7th ed., 2010).			
Stage 0	Tis	N0	M0
Stage I	T1	N0	M0
Stage II	T2	N0	M0
Stage III	T3	N0	M0
Stage IVA	T4	N0	M0
	Any T	N1	M0
IVB	Any T	Any N	M1

TNM staging for perihilar bile duct tumors (7th ed., 2010)			
Stage 0	Tis	N0	M0
Stage I	T1	N0	M0
Stage II	T2a-b	N0	M0
Stage IIIA	T3	N0	M0
IIIB	T1-3	N1	M0
Stage IVA	T4	N0-1	M0
IVB	Any T	N2	M0
	Any T	Any N	M1

### Cell lines culture and reagents

HUCC-T1, TFK-1, and EGI-1 (Riken BRC Cell Bank (Tsukuba, Ibaraki, Japan), German Collection of Microorganisms and Cell Cultures (DSMZ, Braunschweig, Germany)) were used for in vitro experiments. All cell lines were cultivated in RPMI-1640 medium supplemented with 10 % fetal bovine serum, 100 U/mL penicillin, and 100 µg/mL streptomycin in 5 % CO_2_ at 37 °C. Tivantinib (ARQ197, Selleck Chemicals, USA), Apitolisib (GDC-0980, RG7422, Selleck Chemicals, USA), and Refametinib (RDEA119, Bay 86-9766, Selleck Chemicals, USA) were dissolved in 100% dimethyl sulfoxideand stored at −20 °C. Recombinant human HGF was purchased from Sigma-Aldrich, Munich, Germany.

### Cell viability assay

Cells were seeded onto 96-well plates at various cell densities to avoid overgrowth (8 × 10^3^/well) and were treated with increasing concentrations of Tivantinib or a combination of Apitolisib and Refametinib. To investigate the effect of Tivantinib or the combined treatments on cell viability at different concentrations, the cells were kept in culture for 1, 2, 3, and 6 days. At the time points 1, 2, and 3 days, 20 μL of MTT (3-(4,5-dimethylthiazol-2-yl)-2,5-diphenyltetrazolium bromide, 5 mg/mL, Sigma-Aldrich, Munich, Germany) solution was added to each well and incubated for another 4 h, after which the medium was carefully discarded. Formazan (MTT metabolic product) was resuspended in 200 μL of 2-propanol (VWR, Darmstadt, Germany). The plate was placed on a shaker at a speed of 550 r.c.f. for 30 min. Then, the optical density was read at 570 nm using a Biochrom Anthos 2010 microplate reader (Biochrom Ltd, Cambridge, UK). The cell viability after 6 days of treatment with Tivantinib was evaluated using a resazurin-based PrestoBlue reagent (Invitrogen, Carlsbad, CA, USA). As metabolically active cells are capable of reducing the PrestoBlue reagent, the colorimetric changes can be used as an indicator to quantify cell viability in cultures. Cell viability was measured according to the manufacturer’s instructions. Briefly, the PrestoBlue solution (22 µL) was added into each well after 2 h of incubation for the 6-day treatment condition, plates were then placed back into the incubator for a further 2 h of incubation, after which absorbance was measured at 570 nm for excitation and 600 nm for emission.

### Colony-formation assay

Cells were trypsinized and plated in 6-well plates containing 200 cells per well. After overnight attachment, the cells were exposed to treatment conditions for 48 h. The media was replaced with fresh media and then the plates were maintained at 37 °C. Fourteen days later, the cells were fixed and stained using crystal violet (20% ethanol and 0.5% crystal violet in water). The number of colonies, defined as > 50 cells/colony, were counted. Triplicate wells were set up for each condition under investigation.

### Flow cytometry

Cells (1 × 10^5^ to 2 × 10^5^) were seeded in six-well plates and treated after being left to incubate overnight. Fluorescence-activated cell sorting (FACS; CellQuest analysis program, BD Biosciences, Heidelberg, Germany) was performed to detect the sub-G1 cell fraction. This was done to determine apoptosis and the respective phases of the cell cycle after propidium iodide (PI) staining as previously described^27^. In addition, apoptosis was assessed morphologically using Hoechst 33342 staining and fluorescence microscopy.

### Immunofluorescence cytochemistry

Cells seeded on cover slides were fixed with ice-cold methanol. After blocking, the cells were incubated at 37 °C for 45 min with the primary antibody. An antibody diluent with background reducing components from Dako (Glostrup, Denmark) was used to dilute the primary antibodies and as a negative control. After five washes in PBS, the cells were incubated with the fluorochrome-conjugated secondary antibody at 37 °C for 45 min before being washed again with PBS. Nuclear staining was achieved with DAPI (Sigma-Aldrich, Munich, Germany). Finally, cover slides were mounted in a fluorescence mounting medium obtained from Dako.

### siRNA transfection

For c-jun N-terminal protein kinase (JNK) and c-jun knockdown in human CC cell lines, the following pre-designed and pre-validated small interfering RNAs (siRNAs) were purchased from Cell Signaling Technology: SignalSilence^®^ SAPK/JNK siRNA II (catalog number 6233) and SignalSilence^®^ c-jun siRNA II (catalog number 6204). HuCC-T1 and EGI-1 cell lines were transfected with siRNA using Lipofectamine® 2000 Transfection Reagent (Thermo Scientific, Rockford, IL, USA) as per the supplier’s protocol. Transfection efficiency was evaluated after 24, 48, and 72 h using western blotting. The apoptotic effect of Tivantinib on human CC lines after siRNA transfection was detected using MTT assay and FACS.

### Western blotting

Cells were lysed using RIPA buffer (Sigma-Aldrich, Munich, Germany) for 10 min on ice, followed by centrifugation at 16,110 × g for 15 min at 4 °C. The supernatant was collected and the protein concentration was determined using a BCA™ Protein Assay Kit (Thermo Scientific, Rockford, IL, USA). Whole cell extracts (20 μg) were heated with LDS sample buffer (Invitrogen, Carlsbad, CA, USA) at 70 °C for 10 min, separated using SDS-polyacrylamide gel electrophoresisin 4–12 % Bis-Tris gel (Invitrogen, Carlsbad, CA, USA), and transferred to the nitrocellulose membrane (Bio-Rad, Hercules, CA, USA). After blocking for 1 h, the membrane was incubated overnight with primary antibodies at 4 °C. Subsequently, the membrane was probed using horseradish peroxidase-conjugated secondary antibody for 1 h at room temperature. The bands were visualized using a West PICO chemiluminescent substrate (Thermo Scientific, Rockford, IL, USA) and photographed using an image acquisition system (Vilber, Eberhardzell, Germany).

### Antibodies

The following antibodies were used: Actin, p-Met, c-MET, BCL-XL, FADD, p-Bad, bim, cleaved caspase-8, cleaved caspase-3, Phospho-p44/42 MAPK (Erk1/2), p44/42 MAPK (Erk1/2), p-c-jun, p-AKT, AKT, p-Raf, c-Raf Phospho-MEK1/2, MEK1/2 (Cell Signaling, Frankfurt, Germany); caspase-8, JNK1, and p-JNK (Santa Cruz, Heidelberg, Germany); CK19, c-jun, and p-c-jun (Abcam, Cambridge, UK); c-MET neutralizing antibody (Sigma-Aldrich, Munich, Germany); and the secondary antibodies for goat anti-rabbit and goat anti-mouse (LI-COR Biosciences GmbH, Bad Homburg, Germany).

### Statistical analysis

The IC_50_, *t*-test, and one-way analysis of variance were calculated using IBM SPSS Statistics 24.0. The figures were plotted using GraphPad Prism 6.0. The values obtained are the mean (± SEM) of three replicates. Statistical difference was considered significant when *p* ≤ 0.05.

## Results

### c-MET is aberrantly expressed in tumor tissue when compared with the corresponding non-tumor tissue

**Fig. 1:**
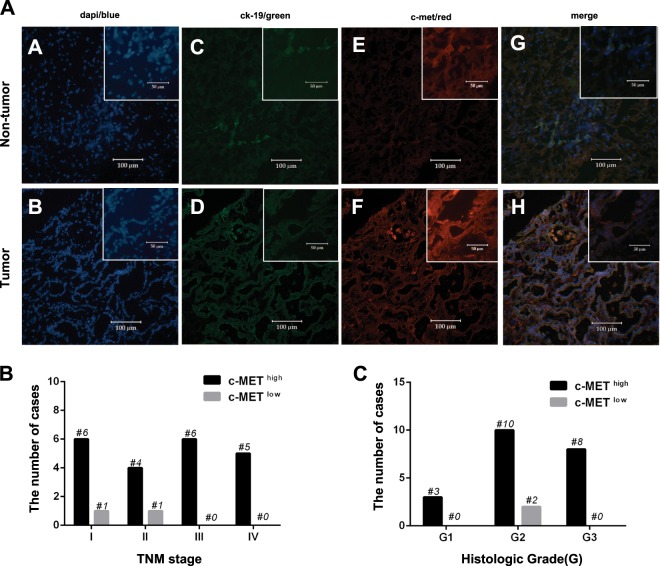
Expression of c-MET in primary CC patients. **a**, **b** DAPI staining in tumor tissue and the corresponding non-tumor tissue. **c**, **d** CK19 was stained to label the lining of the gastroenteropancreatic and hepatobiliary tracts. **e**, **f** Higher c-MET expression was detected in tumor tissues, but not in the corresponding non-tumor tissues. **g**, **h** Merged images. Magnification for A-H: × 20 and × 40 (insets)

To validate the activation of c-MET signaling in CC carcinogenesis, the activation of c-MET expression was measured in 23 corresponding tumor- and non-tumor tissue samples taken from patients with iCC and pCC. Expression of c-MET in both the tumor- and the corresponding non-tumor tissues is presented in Fig. 1a. In comparison with non-tumor tissues, c-MET was overexpressed in 91.3% of tumor tissue samples (21/23, *p* < 0.01). In each TNM stage or tumor histological grade, there were more patients who had a greater expression of c-MET in the tumor tissue than in the non-tumor tissue (Fig. 1b, c).

### Notable losses in cell viability and colony-forming capability were caused by Tivantinib in human CC cell lines

**Fig. 2:**
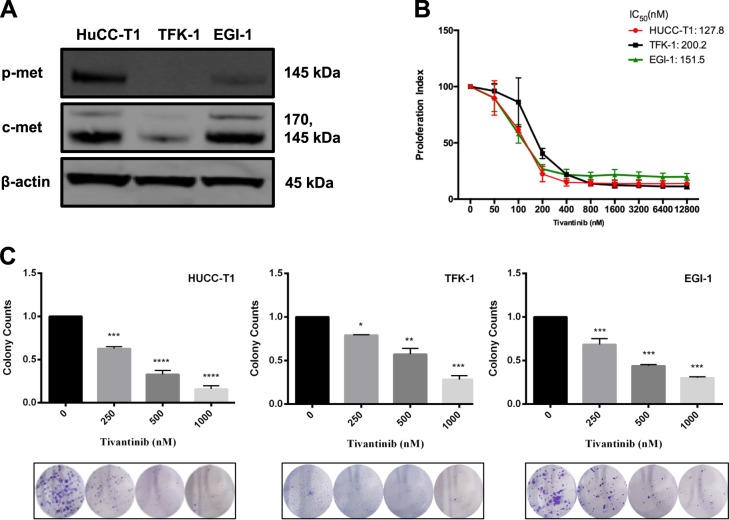
Blocking of c-MET reduces cell viability and colony formation in CC cell lines. **a** Western blotting analysis of c-MET and p-MET in the indicated cell lines. **b** PrestoBlue cell viability reagent represents the effect of increasing concentrations of Tivantinib on cell viability. **c** Colony-forming assay showing representative figures of colonies in each cell lines under Tivantinib treatment. Bar graphs quantify the results as mean and SD. ****p, ***p, and **p < 0.01, and *p < 0.05 vs. the control cells

Tivantinib treatment was shown to cause a dose-dependent loss in cell viability with IC_50_ values in HuCC-T1(127.8 nM), TFK-1(200.2 nM), and EGI-1(151.5 nM) (Fig. 2b). This matched the various naive levels of c-MET expression in these cell lines, which had been obtained using western blotting (Fig. 2a). The cell lines expressing high c-MET showed more sensitivity to the c-MET inhibitor Tivantinib. Likewise, colony-forming assays further confirmed a reduction in the number and size of colonies in the cells treated with Tivantinib (Fig. 2c).

### Tivantinib application inhibited CC growth by inducting apoptosis independent of c-MET downstream pathways

**Fig. 3:**
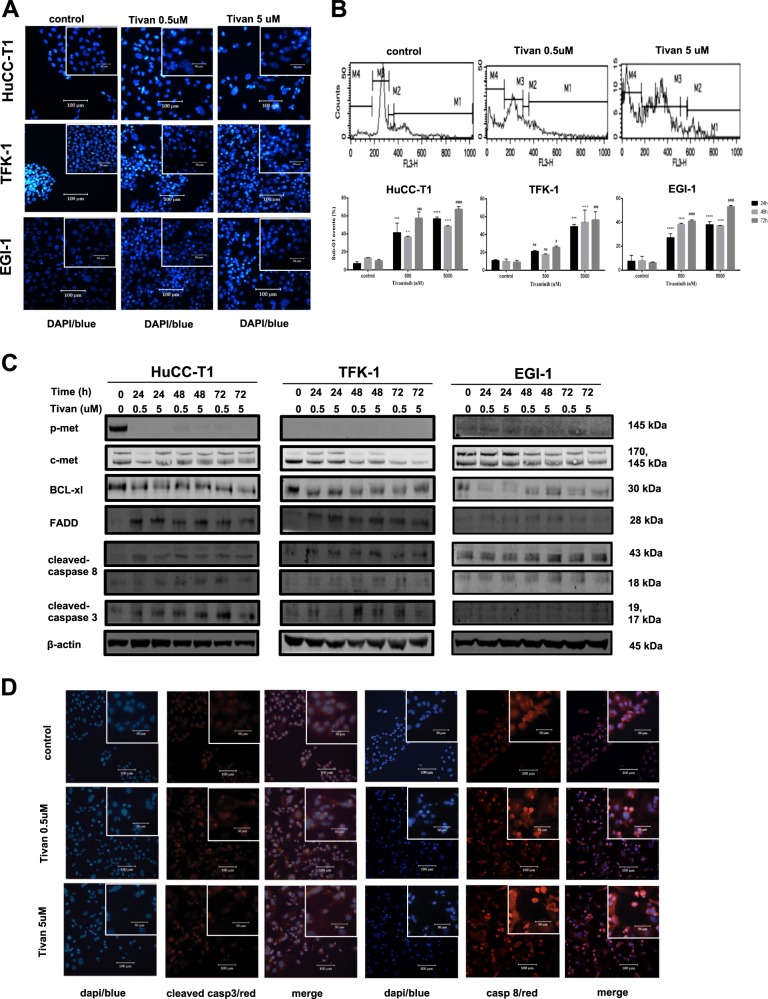
Inhibition of c-MET signaling by Tivantinib results in induction of intrinsic and extrinsic apoptosis. **a** Fluorescence microscopy features after DPAI staining showing nuclear fragmentation in CC cells treated by Tivantinib (0.5 and 5 µM). Magnification: × 20 and × 40 (insets). **b** Apoptosis was quantified via staining with propidium iodide (PI) using flow cytometry. HuCC-T1, TFK-1, and EGI-1 cells were treated with Tivantinib (0.5 and 5 µM) or DMSO for 24, 48, and 72 h. ****p, ***p, **p, ^####^*p*, ^###^*p*, ^##^*p*, ^++++^*p*, ^+++^*p*, ^++^*p* < 0.01, *p, ^#^*p*, and ^+^*p* < 0.05 in comparison with the control cells. **c** Reduction of p-MET, c-MET, and BCL-XL, as well as activation of FADD, caspase-3, and caspase-8, were assessed using western blotting. For this experiment, CC cells were treated with Tivantinib 0.5 and 5 µM for 24, 48, and 72 h. **d** Immunofluorescence cytochemistry analysis of cell apoptosis induced using Tivantinib 0.5 and 5 µM with 24 h exposure in HuCC-T1 cell line. The nuclei were stained with DAPI (blue fluorescence); caspase-8 and cleaved caspase-3 were detected using a Cy3-conjugated secondary antibody (red fluorescence). Merged images show the expression of caspase-8 and cleaved caspase-3 on the background with DAPI. Magnification: × 20 and × 40 (insets)

To evaluate the mechanism by which Tivantinib causes loss of viability in human CC cell lines, cell apoptosis DAPI staining, several parameters were investigated, including sub-G1 cell fraction detection during FACS analysis after PI staining, the presence of apoptotic proteins via western blotting experiments, and immunofluorescence cytochemistry. This was based on recent evidence in which apoptosis was shown to be strongly induced in the presence of Tivantinib^27–29^. Induction of apoptosis was observable at Tivantinib concentrations of 500 and 5000 nM. In addition, cells showed apoptotic features, such as DNA fragments, after 72 h of incubation during DAPI staining (Fig. 3a). FACS indicated an increase in sub-G1 events in both a time- and dose-dependent manner after Tivantinib application (Fig. 3b). Cleaved caspase-3, cleaved caspase-8, and FADD were highly expressed, whereas BCL-xl, p-Met, and c-MET were downregulated (Fig. 3c). Upregulation of activated caspase-3 cleavage and caspase-8 was confirmed using immunofluorescence cytochemistry (Fig. 3d, Supplementary Figures 1A-B).

To further investigate the pro-apoptotic mechanism of Tivantinib, two main downstream c-MET pathways were analyzed: the Pi3k/Akt/mTOR and Ras/Raf/MEK/ERK pathways. Using western blotting analysis, high expression of key proteins from both pathways were detected after the addition of Tivantinib (Supplementary Fig. 2A-B). Similarly, immunoblotting results were also observable in the pancreatic cancer cell line Capan-1 and the hepatocellular carcinoma cell line Huh7 in the presence of Tivantinib (Supplementary Figure 2C). Downregulation of the Pi3k/Akt/mTOR pathway by the Pi3k inhibitor Apitolisib or the Ras/Raf/MEK/ERK pathway by the MEK inhibitor Refametinib did not enhance the anti-tumor effect Tivantinib had on the viability of CC cells. This implied that the pro-apoptotic activity of Tivantinib was independent and unaffected by any blockage of c-MET downstream pathways.

### The synergistic activation of the JNK/c- jun pathway contributed to the induction of apoptosis

**Fig. 4:**
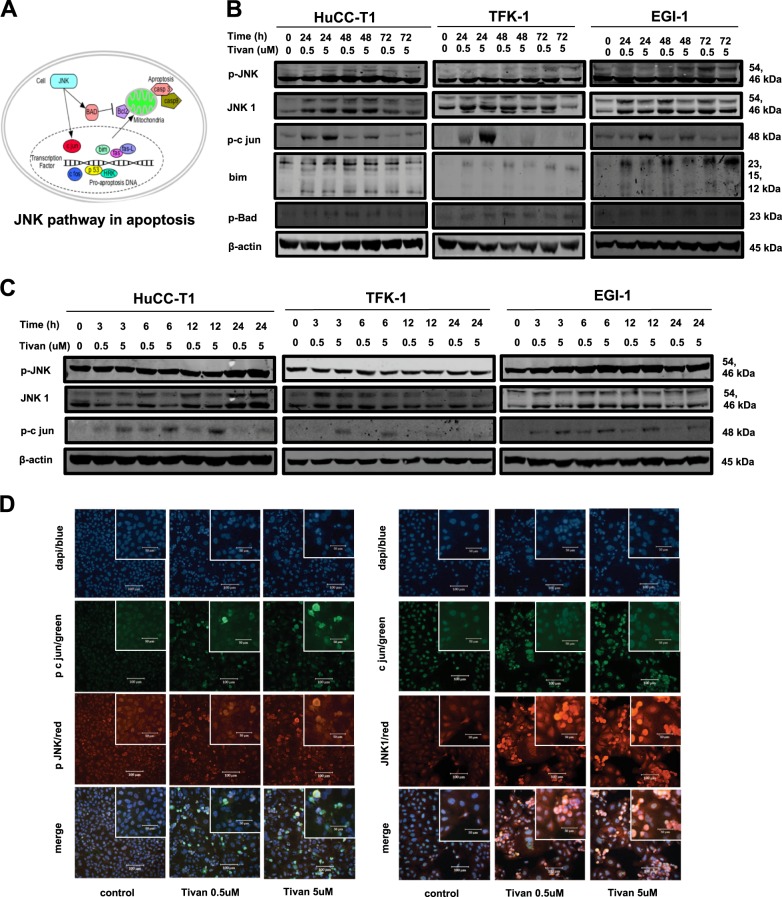
JNK/c-jun pathway is activated simultaneously by Tivantinib. **a** The role of JNK/c-jun pathway in cell apoptosis. **b** JNK and its downstream proteins, p-JNK, JNK1, p-c jun, bim, and p-bad were analyzed using immunoblotting. CC cell lines were treated with Tivantinib 0.5 and 5 µM for 24, 48, and 72 h. **c** In Tivantinib (0.5 and 5 µM) short time exposure, the changes of p-JNK, JNK1, and p-c jun were evaluated using immunoblotting. The exposure times were 3, 6, 12, and 24 h. **d**–**f** Fluorescence microscopy features the localization of p-c jun/p-JNK and c-jun/JNK1 in HuCC-T1 cell. Magnification: × 20 and × 40 (insets)

The JNK/c-jun pathway is known to have a key role in the apoptosis process in many treated tumors^30–33^. As shown by the increasing expression of phosphorylated and total JNK and its downstream proteins such as p-c jun, Bim, and p-Bad in western blotting analysis exposed at 24, 48, and 72 h after the addition of Tivantinib, activation of the JNK/c-jun pathway contributed to the synergistic induction of apoptosis by Tivantinib in CC cells (Fig. 4a, b). In short time expose, Tivantinib had weak effect on the induction of the p-JNK expression but caused high JNK and p-c-jun expression (Fig. 4c). Moreover, in immunofluorescence cytochemistry analysis, the localization of upregulated c-jun at phosphorylated and total level, and in correspondence with the JNK/p-JNK was demonstrated (Fig. 4d, Supplementary Figures 3A-B).

**Fig. 5:**
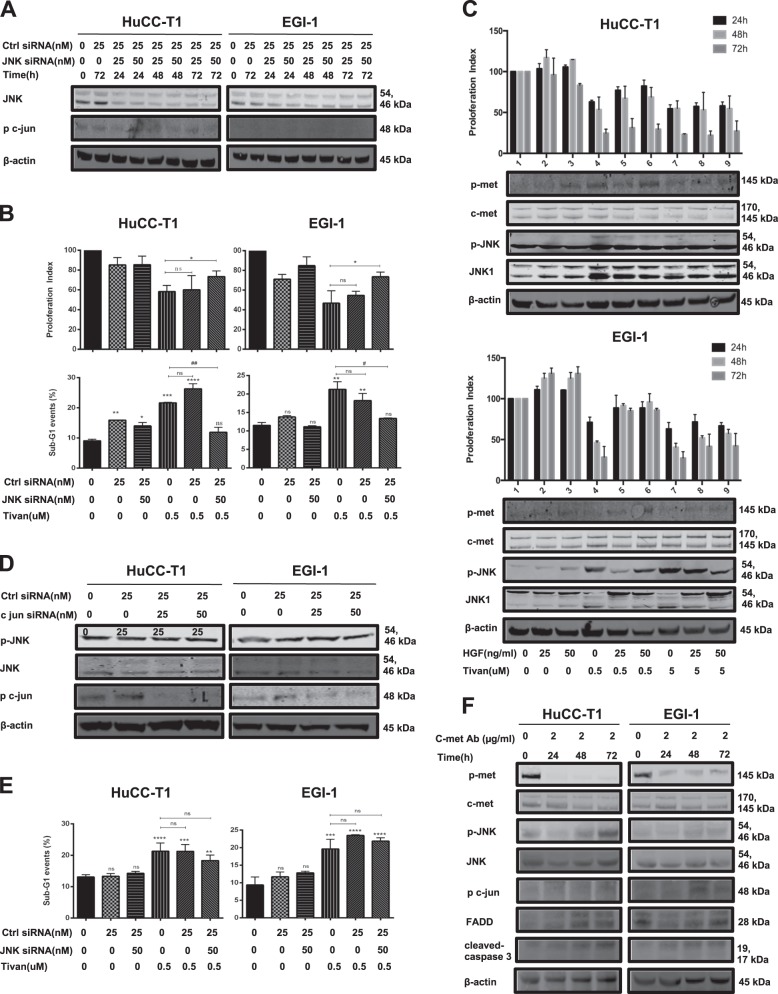
The synergistic activation of JNK/c-jun pathway plays a functional role in determining the apoptotic effect of Tivantinib. **a** Effect on the expression of JNK and c-jun by co-transfection of JNK-specific siRNA oligonucleotide sequences (each at the concentration of 25 and 50 nM) in HuCC-T1 and EGI-1 cell were assessed by western blotting; medium or non-coding siRNA (Ctrl-siRNA) were used as control. **b** Cell viability after transfection of siRNA targeting JNK in HuCC-T1 and EGI-1 cell. *p < 0.05 in comparison with cells treated by Tivantinib 0.5 µM for 24 h. FACS analysis of apoptosis ****p, ***p, **p < 0.01, and *p < 0.05 in comparison with cells with medium or with non-coding siRNA transfection. For this experiment, Tivantinib 0.5 µM was added into cell culture for 3 h after 24 h JNK siRNA transfection. **c** Cell viability analysis of HGF (25 and 50 ng/mL) stimulated cells treated by Tivantinib. p-MET, MET, p-JNK, and JNK1 were evaluated by western blotting in the same condition. HFG stimulated HuCC-T1 and EGI-1 cell were incubated with HGF for 3 h before being added to Tivantinib 0.5 and 5 µM for 24, 48, and 72 h. **d** Effect of c-jun silencing by specific siRNA in CC cells. **e** FACS analysis of apoptosis after transfection of siRNA targeting c-jun.****p, ***p, **p < 0.01, and *p < 0.05 in comparison with cells with medium or with non-coding siRNA transfection. **f** Western blotting analysis of p-MET, MET, p-JNK, JNK1, p-c jun FADD, and cleaved caspase-3 express in HuCC-T1 and EGI-1 cell treated by c-MET neutralizing antibody for 24, 48, and 72 h

To further assess the role of the JNK/c-jun pathway in underlying apoptosis processes, siRNA transfection targeting JNK and c-jun was conducted. As shown in Fig. 5a, b, silencing JNK by simultaneous co-transfection of specific siRNA sequences in HuCC-T1 and EGI-1 cells led to a decrease in Tivantinib-induced apoptosis over the course of 24 h. Competitive binding of the c-MET receptor via stimulation with HGF promoted CC cell growth by increased expression of the c-MET pathway. Simultaneously, the anti-tumor effects of Tivantinib antagonized due to decreased expression of the JNK pathway (Fig. 5c). However, no significant decrease in sub-G1 events during the knockdown of c-jun was observed (Fig. 5d, e). Next, c-MET neutralizing antibody was introduced to validate the role of JNK/c-jun pathway in current study (Fig. 5f). Similar to Tivantinib, blocking the c-MET receptor at 24, 48, and 72 h caused upregulated expression of Cleaved caspase-3 and FADD, and activation of JNK/c-jun pathway.

### Low expression of JNK1 in tumor tissue in CC patients

**Fig. 6:**
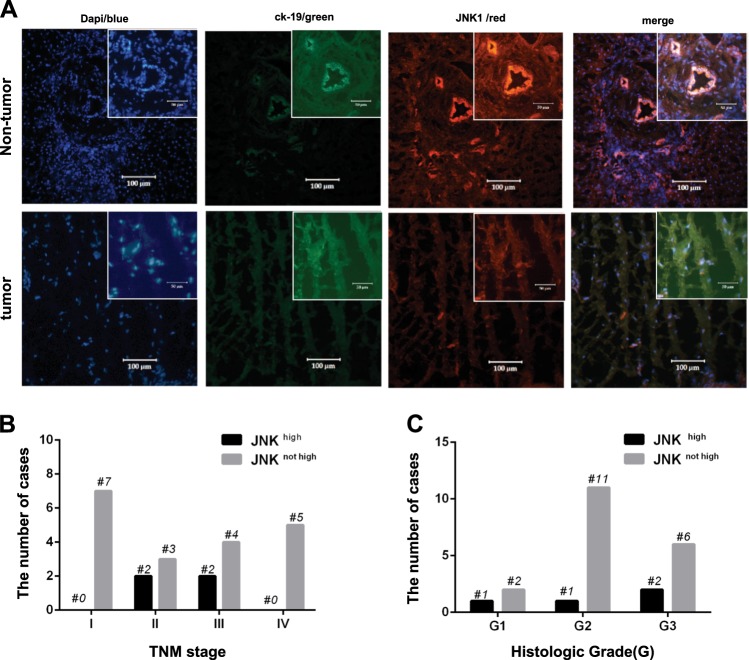
Expression of JNK1 in primary CC cases. **a** Fluorescence immunohistological images of JNK1 expression in non-tumor tissue and the corresponding tumor tissue. The nuclei were stained with DAPI (blue fluorescence), the lining of the gastroenteropancreatic, and hepatobiliary tracts were labeled with CK19 (green fluorescence), and JNK was incubated with Cy3-conjugated secondary antibody (red fluorescence). Merged pictures show the expression of JNK in the biliary system with DAPI background. Magnification: × 20 and × 40 (insets). **b** c-MET and JNK expression in tumor tissues and TNM staging. **c** c-MET and JNK expression in tumor tissues and tumor histological grade

High expression of JNK1 was detected in tumor tissue samples compared to corresponding non-tumor tissue in 17.4% of patients (4/23, *p* < 0.01) (Table 2). However, JNK1 was overexpressed in non-tumor tissues of most patients (16/23 patients). No difference was detected between the non- and tumor tissues in three patients (Fig. 6a). There was no difference in the expression of JNK1 in each TNM stage subgroup or between the various tumor histological grade subgroups (Fig. 6b and Table 2, *p* > 0.05).

**Table 2:** The expression of c-MET and JNK in the tumor tissues compared with corresponding non-tumor tissues

Characteristic		Group (*N* = 23)	*p*	TNM stage (*N* = 23)				*p*	Histologic Grade (*N* = 23)			*p*
				I	II	III	IV		G1	G2	G3	
c-MET	High expression	21(91.3%)	<0.01	6	4	6	5	0.546	3	10	8	0.366
	Low expression	2(8.7%)		1	1	0	0		0	2	0	
JNK	High expression	4(17.4)	<0.01	0	2	2	0	0.147	1	1	2	0.463
	Not high expression	19(82.6)		7	3	4	5		2	11	6

## Discussion

c-MET overexpression is well reported in multiple carcinomas^10,34,35^. In the present study, c-MET overexpression was found in CC tumor tissues compared with non-tumor tissues. It is hypothesized that c-MET might be a promising therapeutic target in CC treatment.

A robust pipeline of high-quality inhibitors targeting different aspects of c-MET activation is currently being investigated in phase I, II, and III clinical trials across multiple tumor types. Preliminary data demonstrate promising clinical activity of these agents, along with an acceptable toxicity profile^20–22^. In the present study, Tivantinib, a non-selective Met inhibitor that stabilizes its non-phosphorylated inactive conformation, was used to inhibit the c-MET expression in vitro^36,37^. However, there are conflicting clinical trial results. In some cases, Tivantinib failed two phase III studies involving second-line treatment of Met-high, advanced, hepatocellular carcinoma (METIV-HCC/JET-HCC), despite its success during phase II studies. The reasons for this might be multifactorial and discussion centers around trial design, inadequacies in the selection of patients according to tumor c-MET status, unclear definition for primary c-MET expression as biomarker for treatment, the inevitable upregulation of c-MET, which had been exposed during first-line treatments such as Sorafenib, and differences in implementation of Tivantinib during studies^22,38–42^. A recent systematic review and meta-analysis on the efficacy and safety of Tivantinib for the treatment of solid tumors concluded that Tivantinib prolonged the progression-free survival, but not the overall survival in patients^43^. This was similar to the results of a phase III study involving epidermal growth factor receptor (EGFR)-mutant non-small cell lung cancer^44^. So far, there is limited information about Met inhibition in CC and no evidence on the efficacy of Tivantinib in CC.

The study demonstrated that targeting c-MET using Tivantinib led to a dose- and time-dependent decrease in the growth, viability, and colony formation in human CC cell lines. Compared with TFK-1, a cell line with low baseline levels of c-MET expression, HuCC-T1 and EGI-1, both of which had relatively high baseline levels of c-MET expression, were found to be more sensitive to Tivantinib. This indicated that Tivantinib inhibited the activation of c-MET signaling and is also involved in the progression of CC, as it effectively inhibits both the full and the phosphorylated form of c-MET. Further, based on recent evidence on its apoptosis-inducing ability, Tivantinib was shown to cause cleavage of Procaspase-3 and -8 followed by the FADD. Cleavage of Caspase-8 was a hallmark of extrinsic apoptotic pathway activation, whereas cleavage of Caspase-3 represented the intrinsic apoptosis involved^45^.

c-MET engagement participates in multiple transduction pathways^13,17–19^. Different downstream targets of the c-MET signaling pathway were analyzed to investigate Tivantinib’s pro-apoptotic mechanisms. This is in contrast to previous studies with c-MET inhibitors^46–48^ in which high expression of p-AKT, AKT, p-raf, c-raf, p-ERK, and ERK after 24 h of exposure to Tivantinib was observed and confirmed in CC cell lines, the pancreatic cancer cell line Capan-1, and the hepatocellular carcinoma cell line Huh7. However, in previous studies with c-MET inhibitors, combination with the Pi3k inhibitor Apitolisib or the MEK inhibitor Refametinib did not affect the anti-tumor efficacy of Tivantinib. This indicated that activation of c-MET downstream pathways, the Pi3k/Akt/mTOR pathway, and the MAPK/ERK pathway seemed to have a marginal role in triggering apoptosis. As both the intrinsic and extrinsic apoptotic pathways were activated in CC cells during Tivantinib treatment, cell apoptosis related to pathways were assessed. Data indicated that Tivantinib activated the JNK/c-jun pathway and induced CC cells apoptosis. The data on JNK was also confirmed by blocking c-MET receptor with neutralizing antibody. Apoptosis was reduced via a decrease in JNK through siRNA transfection or competitive binding of the c-MET receptor by HGF, but not via the silencing of c-jun. The JNK/c-jun pathway could be activated using multiple factors and c-jun activation could be independent or dependent on this pathway^49,50^. Present results indicated that the JNK/c-jun pathway might be involved in the synergistic apoptotic effect exhibited by Tivantinib; among others, it was JNK, rather than its downstream protein c-jun, which had a role in Tivantinib-induced apoptosis. Tivantinib might antagonize the effect of c-MET down-mediated expression of JNK to proceed the apoptotic pathway, the effect of c-jun might not be key role during this process. This was in agreement with previous studies in which c-jun was not essential for apoptosis^51,52^. In contrast, the JNK is a subfamily of the MAPK superfamily, which specifically phosphorylates the transcription factor c-Jun on its N-terminal transactivation domain at two serine residues Ser63 and Ser73^53^. JNK is a key regulator in many cellular events. Apoptosis mediated by the JNK/c-jun pathway was influenced by the cell type, the duration of stimulation, or its upstream and downstream signal pathways. The JNK/c-jun pathway could activate or inhibit apoptosis^50,54^. The present study confirmed that when exposed to Tivantinib, activated JNK was responsible for caspase-3 activation, followed by the downstream proteins p-c jun, bim, and p-Bad.

In previous clinical trials, high c-MET expression was described as an incidental biomarker of tumor sensitivity to the anti-tumor activity of Tivantinib^38,41^. Here, it was demonstrated that Tivantinib also activated JNK and its pathway. JNK, in addition to MET overexpression, could be a potential predictor of Tivantinib efficacy in CC. Therefore, the expression of JNK1 in non-tumor and the corresponding tumor region from patients with CC was detected. There is evidence that JNKs, especially JNK1, had a critical role in death receptor-initiated extrinsic and mitochondrial intrinsic apoptotic pathways^30–33,55–57^. Based on this, it could be hypothesized that CC arising from epithelial origin may already lose the ability to quickly renew epithelial through JNK-mediated cell-programmed death due to tumorous immortality and heterogenesis. In this study, JNK1 was overexpressed in non-tumor tissues compared with the corresponding tumor tissues. There was no difference in the expression of JNK regarding each TNM stage and tumor histological grade. This illustrated that JNK, similar to c-MET, may participate in each developmental stage of cholangiocarcinogenesis and could be a possible alternative predictor for Tivantinib treatment. However, to prove this hypothesis, additional research is needed to define the optimal selection strategy for patients based on the JNK status combined with/without c-MET expression for Tivantinib treatment in CC. Moreover, further investigations are also warranted to demonstrate the relationships in the JNK and c-MET pathway, as well as other possible pathways involved in regulating cell apoptosis in Tivantinib treatment.

In summary, inhibition of c-MET could be a possible alternative approach for the treatment of human CC, for which Tivantinib may an effective inhibitor. The synergistic activation of the JNK/c-jun pathway contributed to elevated apoptosis in CC cells through the treatment with Tivantinib. The data presented indicate great therapeutic potential for CC treatment with c-MET inhibition.

## Supplementary information

Supplemental Figure 1

Supplemental Figure 2

Supplemental Figure 3
