# Supplementary material for: Targeting c-MET by Tivantinib through synergistic activation of JNK/c-jun pathway in cholangiocarcinoma
Source: Cell Death Dis. 2019 Mar 8;10(3):231. doi: 10.1038/s41419-019-1460-1 (PMC6408560; doi:10.1038/s41419-019-1460-1)

S Fig 1:Immunofluorescence cytochemistry analysis of Tivantinib induced cell apoptosis in TFK-1 and EGI-1 cell lines

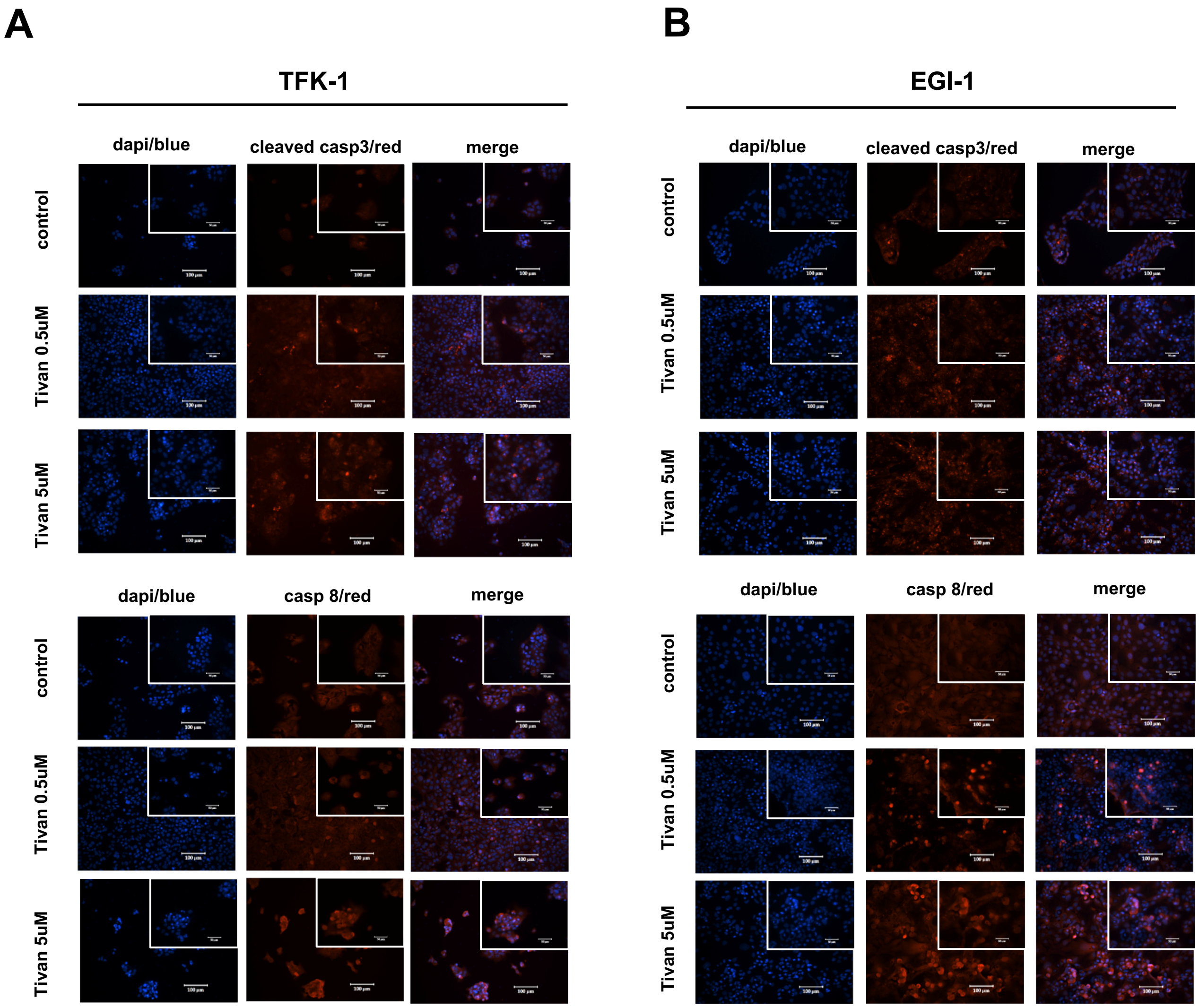

Supplement: Supplementary file 1 — Supplemental Figure 1 [file 41419_2019_1460_MOESM1_ESM.pdf]
