# Supplementary material for: Targeting c-MET by Tivantinib through synergistic activation of JNK/c-jun pathway in cholangiocarcinoma
Source: Cell Death Dis. 2019 Mar 8;10(3):231. doi: 10.1038/s41419-019-1460-1 (PMC6408560; doi:10.1038/s41419-019-1460-1)

# S Fig 2:Tivantinib application inhibits CC growth by inducting apoptosis independent of c-MET downstream pathways

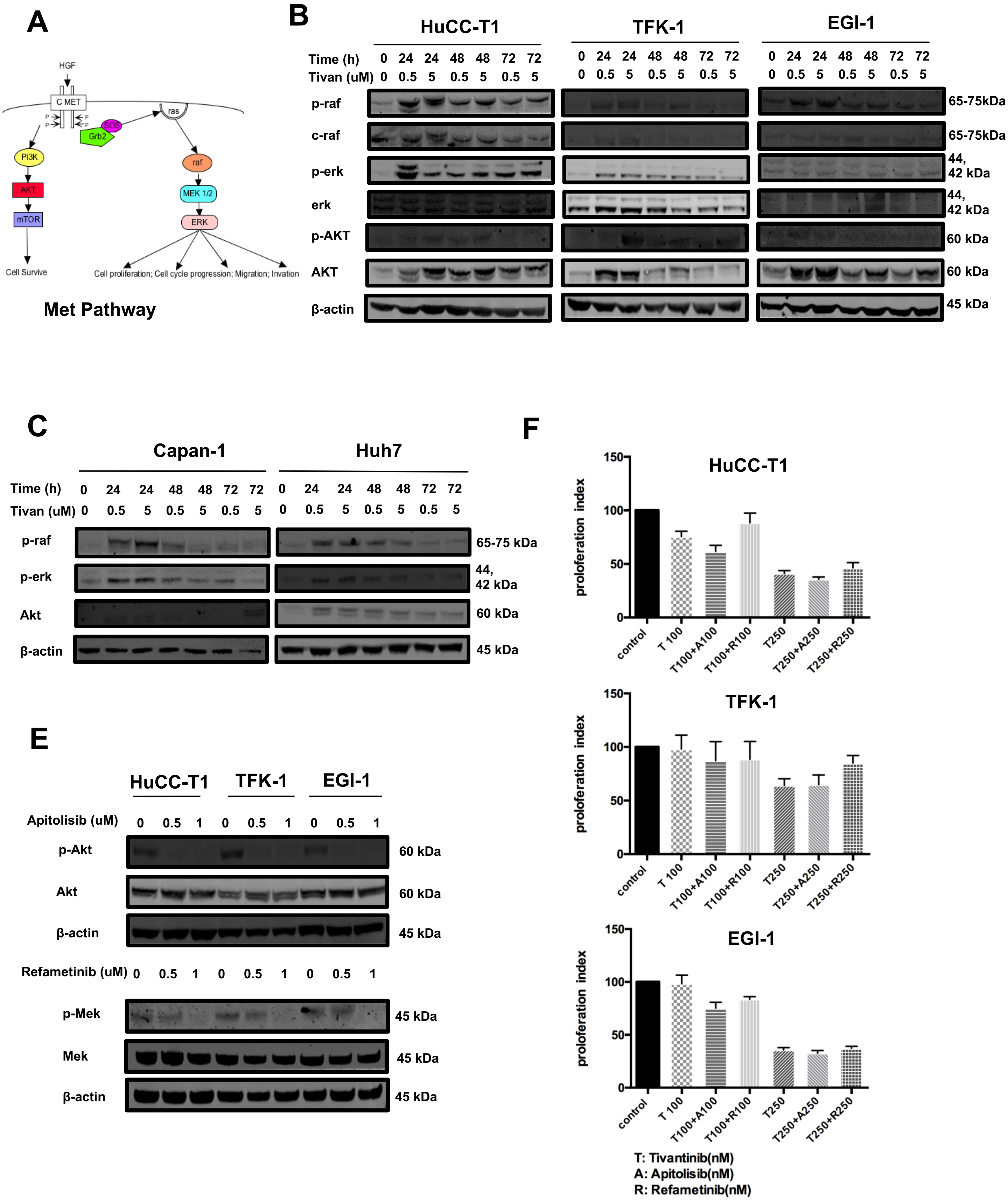

Supplement: Supplementary file 2 — Supplemental Figure 2 [file 41419_2019_1460_MOESM2_ESM.pdf]
